# Supplementary material for: COZOID: contact zone identifier for visual analysis of protein-protein interactions
Source: BMC Bioinformatics. 2018 Apr 6;19:125. doi: 10.1186/s12859-018-2113-6 (PMC5889581; doi:10.1186/s12859-018-2113-6)
Supplement: Supplementary file 2 — Software build. Executable binary file of the software tool. (ZIP 118,784 kb) [file 12859_2018_2113_MOESM2_ESM.zip › cozoid/platform/docs/testng-6.5.1-javadoc.zip!/org/testng/annotations/IParametersAnnotation.html]

IParametersAnnotation (TestNG)


---


|  |  |  |
| --- | --- | --- |
| **Overview** \| **Package** \| **Class** \| **Tree** \| **Deprecated** \| **Index** \| **Help** | |  |
| **PREV CLASS**   **NEXT CLASS** | **FRAMES**    **NO FRAMES**     **All Classes** |  |
| SUMMARY: NESTED \| FIELD \| CONSTR \| METHOD | DETAIL: FIELD \| CONSTR \| METHOD |  |


---


## org.testng.annotations Interface IParametersAnnotation

**All Superinterfaces:**: IAnnotation

---

``` public interface IParametersAnnotation extends IAnnotation ```

Encapsulate the @Parameters / @testng.parameters annotation
Created on Dec 20, 2005

**Author:**
:   Cedric Beust

---

| **Method Summary** | |
| --- | --- |
| `java.lang.String[]` | `getValue()`             The list of variables used to fill the parameters of this method. |

**Method Detail**

### getValue

```
java.lang.String[] getValue()
```

:   The list of variables used to fill the parameters of this method.
    These variables must be defined in your testng.xml file.
    For example

    `@Parameters({ "xmlPath" })  
    @Test  
    public void verifyXmlFile(String path) { ... }` 

    and in testng.xml:

    `<parameter name="xmlPath" value="account.xml" />`


---


|  |  |  |
| --- | --- | --- |
| **Overview** \| **Package** \| **Class** \| **Tree** \| **Deprecated** \| **Index** \| **Help** | |  |
| **PREV CLASS**   **NEXT CLASS** | **FRAMES**    **NO FRAMES**     **All Classes** |  |
| SUMMARY: NESTED \| FIELD \| CONSTR \| METHOD | DETAIL: FIELD \| CONSTR \| METHOD |  |


---
